# Association of Dilated Perivascular Spaces With Lipid Indices in Ischemic Stroke Patients

**DOI:** 10.7759/cureus.28783

**Published:** 2022-09-05

**Authors:** Sevda Diker, Pınar Gelener, Aysegül Erem, Ugurcan Balyemez

**Affiliations:** 1 Neurolgy, Cyprus International University, Nicosia, CYP; 2 Neurology, University of Kyrenia, Kyrenia, CYP; 3 Basic Sciences & Humanities, Cyprus International University, Nicosia, CYP; 4 Radiology, Near East University, Nicosia, CYP

**Keywords:** low-density lipoprotein, high-density lipoprotein, triglyceride, ischemic stroke, lipid profile, perivascular spaces

## Abstract

Background

Dilated perivascular spaces (dPVS) in the basal ganglia are associated with aging, vascular risk factors, and other magnetic resonance imaging (MRI) markers of cerebral small vessel disease (cSVD). While high blood lipids are a well-demonstrated risk factor for large artery atherosclerosis, their role in cSVD remains largely elusive.

Methods

We evaluated lipid profiles, cardiovascular risk factors, and brain MRI findings in patients with ischemic stroke or transient ischemic attack. We analyzed the extent of dPVS, cerebral microbleed (CMB), and cerebral white matter hyperintensities (WMHs) as MRI indices of cSVD and investigated associations of dPVS with lipid parameters and other cSVD indices.

Results

Our study enrolled 173 patients with ischemic stroke or transient ischemic attack. The mean age was 68.38±14.31 (range 35-99) years, and 57.8% (n=100) of patients were male. dPVSwere detected in 97% (n=168) of the patients. Among the whole population, half of the patients (n=87) had moderate to severe dPVS. According to the univariate analysis, age, hypertension, previous antiaggregant and/or anticoagulant use, and the high-density lipoprotein to low-density lipoprotein (HDL/LDL) ratio but not other lipid profiles, cerebral microbleed load, and cerebral white matter hyperintensities severity were found to be positively associated with dPVS number in the basal ganglia. After multivariate logistic regression analysis, only age and WMH severity remained statistically significant.

Conclusions

dPVS are closely associated with other cSVD subtypes and aging. The studied lipid indices were not independently associated with moderate to severe dPVS in basal ganglia in ischemic stroke patients. The association of each lipid and HDL/LDL ratio needs to be further studied with a larger number of participants.

## Introduction

The term cerebral small vessel disease (cSVD) is used to define all the pathological processes that affect small vessels of the brain, including small arteries and arterioles as well as capillaries and small veins [1]. Leukoaraiosis, which refers to neuroimaging abnormalities of the white matter, and silent lacunar infarcts have been the most studied magnetic resonance imaging (MRI) correlates of cSVD [1-3]. 

The fluid-filled spaces that surround small blood vessels in the brain are known as perivascular spaces, which include periarteriolar, pericapillary, and perivenular spaces [4]. Through these spaces, a range of substances can move, and the physiologic role of perivascular spaces relates to the drainage of brain interstitial fluid into perivascular routes, subarachnoid space, and the glymphatic system. They contribute to the brain’s fluid and waste clearance systems and to the pathogenesis of cerebrovascular, neuroinflammatory, and neurodegenerative disorders [5]. While the underlying mechanisms remain unclear, with increasing age and with other features of cSVD [6] and vascular risk factors, especially hypertension [7], perivascular spaces can dilate and become detectable on brain MRI. Dilated perivascular spaces (dPVS) are seen on brain MRI as thin linear or punctate structures (depending on the scan orientation) that have a similar signal to cerebrospinal fluid [8]. Basal ganglia (proximal) dPVS are shown to be more common with vascular dementias and hypertension, while subcortical (distal) dPVS are more common in nonvascular dementias and multiple sclerosis [5].

MRI studies focusing on dPVS started in the mid-2000s [4, 9]. Population-based studies have shown variable degrees of correlation of dPVS with white matter hyperintensities (WMHs) and lacunar infarcts; therefore, dPVS, especially those located in the basal ganglia, have been proposed as an emerging marker for cSVD [8, 10, 11]; however, the pathology of dPVS remains unclear.

On the other hand, high blood lipids are a well-demonstrated risk factor for large artery atherosclerosis [12], although their role in cSVD remains largely elusive. High low-density lipoprotein (LDL) or total cholesterol levels were even found to serve as a protective factor against cerebral microbleed (CMB) formation [13].

dPVS have been demonstrated to be associated with neurological diseases such as stroke, mild cognitive impairment, and dementia involving the vascular subtype [14, 15]. Therefore, it is of clinical importance to clarify the etiopathogenesis and risk factors of dPVS and search for treatable methods. The relationship between MRI-visible perivascular spaces (PVS) and traditional lipid profile differences have yet to be established. We aimed to determine whether lipids, a potentially intervenable target, were associated with dPVS, which in turn may be associated with ischemic stroke. If so, intervening lipid profiles would provide a possible mechanism for reducing dPVS severity, other associated cSVD, and ischemic stroke incidence. 

## Materials and methods

We performed a retrospective cohort analysis of consecutive ischemic stroke or transient ischemic attack (TIA) patients admitted to the University of Kyrenia Hospital in Kyrenia, Cyprus, between January 2018 and July 2021. We retrospectively evaluated the medical records, laboratory test results, and radiological findings of all of the study patients from our database. We only included patients whose records contained adequate demographic, clinical, radiological, and laboratory data. Patients with contraindications to MRI, like an intracardiac defibrillator, cardiac pacemaker, or metallic heart valve, or who were admitted one month after symptom onset were excluded.

Hypertension was defined as having a systolic blood pressure (SBP) of ≥140 mm Hg or diastolic blood pressure (DBP) of ≥90 mm Hg in at least two blood pressure measurements, use of any antihypertensive drug, or a self-reported history of hypertension. Diabetes mellitus (DM) was considered to be present when a person used oral antidiabetic drugs or insulin or when the fasting blood glucose was 126 mg/dl mg/dl. Hyperlipidemia was considered to be present when the person used lipid-lowering drugs or had a total cholesterol level of 220 mg/dl. Atrial fibrillation (AF) was considered to be present if the patient either had a diagnosis of AF, the admission electrocardiogram revealed AF, or the Holter rhythm recording revealed AF or paroxysmal AF. 

Within 24 hours of hospital admission, blood samples drawn for low-density lipoprotein (LDL), high-density lipoprotein (HDL), triglycerides, and total cholesterol after overnight fasting were recorded. The HDL/ LDL ratio was calculated as the HDL measure divided by LDL. 

An experienced neuroradiologist who was blinded to the clinical information retrospectively evaluated images of the T2 series, fluid-attenuated inversion recovery (FLAIR), diffusion-weighted imaging, apparent diffusion coefficient map, and susceptibility weighted imaging (SWI) sequences obtained in the 1.5T MR-system (Magnetom Aera 1.5T, Siemens Healthcare, Erlangen, Germany). Rating dPVS in the basal ganglia was preferred instead of the centrum semiovale (CSO), taking into account the closer relationship with vascular risk factors of dPVS in this region. dPVS was rated on T2 weighted (T2W) images following the method of Potter et al. To avoid rating lacunas or WMH as dPVS, T2 weighted images were cross-checked against FLAIR, and T1 weighted images [16]. The scale ranged from 0 (no dPVS), 1 (mild; 1-10 dPVS), 2 (moderate; 11-20 dPVS), 3 (frequent; 21-40 dPVS) to 4 (severe; >40 dPVS) based on an estimate of the number of PVS seen in the slice considered to have more of them in the basal ganglia. 

CMB was defined as ovoid or rounded signal void spots less than 1 cm on T2* gradient recalled echo (GRE) or SWI sequences. Signal voids caused by sulcal vascular structures, old hemorrhagic cerebrovascular event sequelae, basal ganglion or pineal gland calcifications were excluded.

Evaluation of WMH was made on FLAIR images included in the MRI protocol. The Fazekas score was used to determine the gliosis severity by grading white matter hyperintensities on MRI [31]. WMH severity was graded as: 0 = absent; 1 = punctuate foci; 2 = beginning of confluence of foci; and 3 = large confluent areas.

SPSS version 21 (IBM Inc., Armonk, New York) was used to perform all statistical analyses in this paper. We grouped patients into two according to dPVS severity as dPVS count 10 or fewer (mild, n=86) and >0 (moderate to severe, n=87) [4]. In the preliminary analysis, the Chi-square test was used for the comparisons of demographic characteristics and dPVS groups. For the comparison of the lipid values among dPVS groups, the Mann-Whitney U test was performed since the assumptions of parametric tests were not satisfied. Lastly, to investigate the effects of lipid values and demographic characteristics of the patients on dPVS, a multivariate logistic regression analysis was performed. The independent variables included in the model were stroke age, HDL/LDL ratio, the presence of hypertension, and the use of antithrombotic drugs. The composed model did not suffer from multicollinearity problems. Ninety-five percent confidence intervals (CI) and the odds ratios (OR) were estimated. A p-value less than 0.05 was considered statistically significant. While the continuous variables are presented as means ± standard deviation, the categorical variables are presented as percentages in the manuscript.

## Results

Our study enrolled 173 patients with ischemic stroke or TIA. The characteristics of the study patients are shown in Table 1. 

**Table 1:** Demographic characteristics, radiological features, and lipid profiles of the study patients CMB - cerebral microbleed, dPVS - dilated perivascular space, HDL - high=density lipoprotein, HDL/LDL - high-density lipoprotein to low-density lipoprotein ratio, LDL - low-density lipoprotein, SWI - susceptibility-weighted imaging, Trig/HDL - triglyceride to high-density lipoprotein ratio

Characteristic	n (%)
Age (mean±SD)	68.38±14.31
Time from symptom onset to admission, hour (mean±SD)	18.03±25.8
Sex	
Female	73 (42.2)
Male	100 (57.8)
Hypertension	112 (64.7)
Diabetes mellitus	51 (29.5)
Atrial fibrillation	53 (30.6)
Hyperlipidemia	54 (31.6)
Coronary artery disease	35 (20.2)
Previous TIA/Stroke	39 (22.5)
Antithrombotic use at the time of TIA/stroke	
None	99 (57.2)
Antiplatelet monotherapy	40 (23.1)
Dual antiplatelet	10 (5.8)
Anticoagulant	19 (11)
Anticoagulant plus antiplatelet	5 (2.9)
Basal ganglia dPVS	
None	5 (2.9)
1-10	81 (46.8)
11-20	46 (26.6)
21-40	27 (15.6)
>40	14 (8.1)
Fazekas score	
0	35 (20.4)
1	44 (25.6)
2	47 (27.3)
3	46 (26.7)
FLAIR absent	1 (0.5)
CMB number	
0	111 (64.2)
1-4	37 (21.4)
5 or more	22 (12.8)
SWI absent	3 (1.6)
Large artery atherosclerosis	40 (24)
LDL (mean±SD)	120.51±37.90
HDL (mean±SD)	41.93±11.95
Total Cholesterol (mean±SD)	189.69±45.83
Triglyceride (mean±SD)	159.33±163.17
Triglyceride /HDL (mean±SD)	4.41±5.76
HDL/LDL (mean±SD)	0.39±0.17

The mean age was 68.38±14.31 (range 35-99) years, and 57.8% (n=100) of patients were male. dPVS were detected in 97% (n= 169) of the patients. The prevalence of patients with a dPVS count >10 (moderate to severe dPVS) was 50% (n=87). We compared the lipid profiles of patients with mild dPVS and moderate to severe dPVS (Table 2).

**Table 2:** Univariate analysis for basal ganglia dPVS CMB - cerebral microbleed, dPVS - dilated perivascular space, HDL - high-density lipoprotein, HDL/LDL - high-density lipoprotein to low-density lipoprotein ratio, LDL - low-density lipoprotein, SWI - susceptibility-weighted imaging, Trig/HDL - triglyceride to high-density lipoprotein ratio, TIA - transient ischemic attack * p<0.05

Characteristic	Mild	Moderate to severe	p-value
Age (mean±SD)	63.41±14.85	74.63±7.79	0.00*
Male sex	55 (64%)	45 (51.7%)	0.103
Hypertension	48 (55.8%)	64 (73.6%)	0.015*
Diabetes mellitus	25 (29.1%)	26 (29.9%)	0.906
Atrial fibrillation	24 (28.2%)	29 (34.1%)	0.408
Hyperlipidemia	30 (34.9%)	24 (27.6%)	0.300
Coronary artery disease	13 (15.1%)	22 (25.6%)	0.088
Previous TIA/stroke	18 (20.9%)	21 (24.1%)	0.614
Antithrombotic use at the time of TIA/stroke	28 (32.6%)	46 (52.9%)	0.007*
Fazekas score			
0	32 (37.6%)	3 (3.4)	
1	28 (32.9%)	16 (18.4%)	0.00*
2	16 (18.8%)	31 (35.6%)	
3	9 (10.6%)	37 (42.5)	
CMB			
0	65 (76.5%)	46 (54.1%)	
1-4	16 (18.8%)	21 (24.7%)	0.002*
5 or more	4 (4.7%)	18 (21.2%)	
Large artery atherosclerosis	18 (20.9%)	22 (27.3)	0.346
Lipid profile			
LDL	123.76±40.89	115.41±34.80	0.156
HDL	38.16±10.95	41.57±11.12	0.260
Total Cholesterol	196.18±45.91	184.67±39.015	0.156
Triglyceride	167.78±101.57	147.85±87.9	0943
Trig/HDL	4.99±3.93	3.98±2.81	0.582
HDL/LDL	0.34±0.148	0.40±0.19	0.04*

While the LDL, HDL, total cholesterol, and triglyceride levels were similar between groups, the HDL/LDL ratio was higher in patients with moderate to severe dPVS in the basal ganglia compared to those with mild dPVS (0.40±0.19 vs 0.34±0.148, respectively, p=0.04). Similarly, the age was higher (63.41±14.85 vs 74.63±7.79, respectively p<0.001), and hypertension was more common (73.6% versus 55.8%, respectively, p=0.015) in this group compared to patients with mild dPVS. Although other vascular disorders such as DM, coronary artery disease, previous TIA/ischemic stroke, or atrial fibrillation were seen with similar rates in both groups, the proportion of antiaggregant and/or anticoagulant use at the time of admission was higher among patients with moderate to severe dPVS (52.9% versus 32.6% respectively, p=0.007) (Table 2). 

After logistic regression analysis, stroke age remained the only statistically significant factor for dPVS load (p<0.001). For every year of age, the odds of having moderate to severe PVS increased by a factor of 1.059 (Table 3). 

**Table 3:** Logistic regression analysis for dPVS CMB - cerebral microbleed, HDL/LDL - high-density lipoprotein to low-density lipoprotein ratio, dPVS - dilated perivascular spaces *p<0.05

	OR (95% CI)	p-value
Hypertension	0.885 (0.3-1.656)	0.696
HDL/LDL	4.109 (0.288-21.853)	0.233
Age	1.059 (1.042-1.116)	0.005*
Antithrombotic use	1.233 (0.455-2.375)	0.633
Fazekas score 0	Reference	0.001
Fazekas score 1	1.736 (0.529-5.697)	0.363
Fazekas score 2	5.161 (1.474-18.071)	0.010*
Fazekas score 3	9.68 (2.602-36.017)	0.001*
CMB	0.556 (0.243-1.269)	0.250

When we compared the presence of dPVS with other cSVD markers (Table 2), we found positive associations where the association with WMH was the most prominent. dPVS>10 was significantly more common in patients with Fazekas scores reflecting moderate to severe deep WMH (Fazekas 2-3) compared to mild WMH (Fazekas 0-1) (p<0.001 OR: 8.73 (95%CI: 4.38-17.40)). In multivariate logistic regression analysis, this association between dPVS and WMH remained significant (p<0.001) (Table 3).

According to the univariate analysis, there was a statistically significant association between dPVS and both CMB presence and CMB count (Table 2). However, this association was not significant after multivariate logistic regression analysis (p=0.250 OR: 0.556 (0.243-1.269) (Table 3).

## Discussion

The role of lipids in cSVD remains mostly unclear. Blood lipids are causally involved in the pathogenesis of atherosclerosis. They are a well-established risk factor for large artery atherosclerosis [17], and statins have shown benefits in reducing the risk of both coronary artery disease and stroke [18]. However, the Japan Statin Treatment Against Recurrent Stroke (J-STARS) trial found statins to reduce the recurrence of large artery stroke but not small vessel stroke [19]. 

On the other hand, basal ganglia dPVS, compared to dPVS in CSO, were shown to be more closely associated with underlying vascular disorders, mainly affecting small vessels [5-7]. Here, we explored the association of blood lipid levels with dPVS, a small vessel disease phenotype in patients with ischemic stroke. 

We detected basal ganglia dPVS in 97% (n=169) of the patients, and such a high proportion is probably due to the age distribution of our patients. Among all patients, 65% (n=113) were aged 65 or older. Similar to the literature, we found a statistically significant association between age and dPVS in the basal ganglia [7]. In a meta-analysis including 8395 individuals, the age and perivascular space visibility association were strongest in the basal ganglia compared to the centrum semiovale and hippocampus [15]. 

When we compared vascular risk factors, among DM, AF, coronary heart disease, previous TIA/stroke, and hyperlipidemia, hypertension was the only factor that showed a positive correlation with dPVS. Compatible with our findings, in population-based studies of older adults, perivascular space visibility in the basal ganglia also increased significantly with hypertension, but not with traditional cardiovascular risk factors, such as smoking, being overweight, stroke history, high total cholesterol, and DM [7, 20, 21].

We demonstrated a significant stepwise association of WMH Fazekas scores and a less significant association of CMB with dPVS. Population-based studies have shown variable degrees of correlation of dPVS with MRI markers of cSVD [8, 10, 11]. Many cross-sectional studies have demonstrated that perivascular spaces are associated with WMHs - the more visible perivascular spaces there are, the more severe the WMHs, and WMHs seem to form around perivascular spaces [22, 23]. Our findings regarding the association of dPVS with age, hypertension, and WMH degree could largely be expected, as dPVS in basal ganglia is a specific marker of cSVD.

In our study, we directly compared dPVS load and lipid fractions in ischemic stroke patients. Although high LDL and low HDL are well-established risk factors for large artery atherosclerosis [17], our results could not show a similar association for cSVD. In the literature, the association between lipid profiles and cerebral SVD has mostly been investigated through CMBs, as it is hypothesized that there is an increased risk of intracranial hemorrhage in patients with low cholesterol levels, mainly triglyceride [24]. In many studies, serum cholesterol levels, especially LDL, were found to be inversely related to the presence of cerebral microbleeds [13, 25]. In terms of WMH, a study that analyzed two independent hospital-based acute ischemic stroke patients demonstrated that hyperlipidemia was associated with reduced WMH severity [2]. In another study, hypertriglyceridemia was found to be associated with reduced leukoaraiosis in patients with a small vessel stroke [3] but not other lipid fractions. A recent study among nondiabetic adults investigated the relationship between total cholesterol, LDL, HDL, and moderate to severe dPVS and, like our study, showed nonsignificant after multivariate comparison [26]. 

Although it was a weak association that was not significant after multivariate regression analysis, the HDL/LDL ratio was positively associated with moderate to severe dPVS in the basal ganglia. The HDL/LDL ratio is more illustrative because it describes the effect of both LDL and HDL on dPVS. However, increasing evidence points to the fact that a decreased HDL/LDL ratio increases the risks of atherosclerotic cardiovascular and cerebrovascular diseases [27, 28]. On the other hand, a lower LDL/HDL ratio was linked to worse outcomes at three months (including death, recurrence, and moderate disability) after stroke [29]. In another study, low levels of non-HDL cholesterol and LDL were associated with an increased risk of hemorrhagic transformation after ischemic stroke [30]. However, these studies, including our study, demonstrate associations, not causative relationships, and the mechanisms underlying these associations are still to be identified. Taking into account the higher quality evidence for coronary artery disease or ischemic cerebrovascular disease [17, 18], interventions to improve lipid profiles, such as lowering LDL, should be undertaken. 

The limitations of our study are its retrospective design and a relatively small number of participants. Our results were derived from a patient population enrolled in a single center and should be confirmed in studies with larger populations. Second, we did not obtain information on lipid-lowering therapy prior to stroke onset. 

## Conclusions

dPVS in the basal ganglia are associated with aging and other MRI markers of cSVD, especially WMH. Considering their association with neurological diseases, such as stroke, mild cognitive impairment, and dementia involving the vascular subtype, it is important to clarify the etiopathogenesis and risk factors of dPVS and search for treatable methods. High-quality evidence exists for the association between lipids and large artery atherosclerosis. In terms of cSVD, we could not find a correlation between dPVS load and LDL, HDL, and triglyceride levels. Further studies with larger numbers of participants are needed to examine these associations.
